# Decrease of Fibulin-3 in Hepatocellular Carcinoma Indicates Poor Prognosis

**DOI:** 10.1371/journal.pone.0070511

**Published:** 2013-08-01

**Authors:** Rongzhen Luo, Meifang Zhang, Lili Liu, Shixun Lu, Chris Zhiyi Zhang, Jingping Yun

**Affiliations:** State Key Laboratory of Oncology in Southern China, and Department of Pathology, Sun Yat-sen University Cancer Center, Guangzhou, China; University College London, United Kingdom

## Abstract

Fibulin-3, originally identified in senescent and Werner syndrome fibroblasts, has been implicated in cell morphology, growth, adhesion and motility. Fibulin-3 exhibits both antitumor and oncogenic activities towards human cancers; however, the role of Fibulin-3 in hepatocellular carcinoma (HCC) remains elusive. In this study, we showed that both the mRNA and protein levels of Fibulin-3 were remarkably downregulated in HCC cell lines and fresh tissues. Immunohistochemical data revealed that Fibulin-3 was decreased in tumorous tissues in 67.1% (171/255) of cases compared to the corresponding adjacent nontumorous tissues. The results of statistical analysis indicated that low Fibulin-3 expression, defined by the receiver operating characteristic curve (ROC), was significantly associated with tumor differentiation (*P* = 0.008), clinical stage (*P* = 0.014) and serum AFP levels (*P*<0.01). Furthermore, Kaplan-Meier and multivariate analysis suggested that Fibulin-3 is an independent negative prognostic indicator for both overall (*P*<0.001) and recurrence-free (*P* = 0.036) survival. In addition, an *in vitro* study demonstrated that knockdown of Fibulin-3 by siRNA markedly increased cell viability and promoted cell invasion in HCC cells. Collectively, our data suggest that Fibulin-3 exhibits antitumor effects towards HCC and serves as a biomarker of unfavorable prognosis for this deadly disease.

## Introduction

Hepatocellular carcinoma (HCC) is the fifth most prevalent malignancy diagnosed worldwide [1]. In recent decades, its incidence has been increasing in economically developed regions, including Japan, Western Europe and the United States [2], [3]. Despite advances in chemotherapy, surgical management and the clinical implementation of numerous therapeutic strategies, the mortality rate of HCC remains very high (up to 94%), making HCC the third most common cause of cancer-related death [1], [4], [5]. Etiology studies indicate that infection with a hepatitis virus (HBV or HCV) and the dysregulation of genes involved in cell proliferation are major risk factors for hepatocarcinogenesis [6], [7]. However, the accuracy and reproducibility of markers currently used in the clinic to predict prognosis after surgical resection are unsatisfactory [4]. It is therefore urgent and important to search for potential prognostic biomarkers to improve clinical management of HCC.

Fibulins, a group of secreted glycoproteins, comprise seven members characterized by repeated epidermal growth-factor-like domains and a unique C-terminal fibulin-type module [8]. Fibulins have been implicated in cell morphology, growth, adhesion and motility [8], [9], [10]. The Fibulin-3 gene is located at chromosome 2p16, contains 11 exons, and encodes a 493-amino acid protein with a molecular mass of 54 kD [8]. Fibulin-3 was originally identified in senescent and Werner syndrome fibroblasts [11]. It is highly conserved among different species with 92–94% of amino acids identical in human, rat and mouse [11], [12], [13]. In cancer, Fibulin-3 is differently expressed. Plasma Fibulin-3 levels have been demonstrated to be upregulated in mesothelioma [14]. An increase of Fibulin-3 was also observed in pancreatic cancer [15], cervical carcinomas [16] and malignant gliomas [17]. On the other hand, Fibulin-3 was found to be downregulated in colorectal [18], lung [19], breast [20], prostate [21], and nasopharyngeal carcinomas [22]. Although Nomoto et al. reported that mRNA expression of Fibulin-3 was decreased in methylated HCC cases [23], the protein level and clinical significance of Fibulin-3 in HCC have not been elucidated.

In this study, Fibulin-3 expression was first examined in HCC cell lines and tissue samples. Correlation of Fibulin-3 expression and clinicopathological features of HCC patients was then investigated. The prognostic value of Fibulin-3 in HCC was also evaluated. Moreover, the effects of Fibulin-3 on HCC cell proliferation and invasion were determined *in vitro*. Our data suggest that Fibulin-3 associates with tumor progression and functions as a tumor suppressor in HCC.

## Materials and Methods

### Cell Culture and Transfection

Huh-7, PLC/PRF/5, HepG2 and SK-Hep-1 cells were purchased from American Type Culture Collection (ATCC, Manassas, VA), and cultured in Dulbecco’s modified Eagle’s medium (DMEM) (Gibco, Gaithersburg, MD, USA) containing 10% fetal bovine serum (FBS), 100 mg/ml penicillin, and 100 mg/ml streptomycin. L02, SMMC-7721, Bel-7404, Bel-7402, QGY-7701, and QSG-7703 cell lines were obtained from the Type Culture Collection Cell Bank, Chinese Academy of Science Committee (Shanghai, China) and were cultured in Roswell Park Memorial Institute (RPMI) 1640 with 10% fetal bovine serum (FBS), 100 U/ml of penicillin, and 100 U/ml of streptomycin. All of the cells were incubated in a humidified atmosphere of 5% CO_2_ and 95% air at 37°C. QSG-7703 cells were transfected with Fibulin-3 siRNA using lipofectamine 2000 (Invitrogen, Carlsbad, CA) according to the manufacturer’s instructions with the following target sequences: Fibulin-3 siRNA #1: CACAACGTGTGCCAAGACATA and Fibulin-3 siRNA #2: CACGCAATGCACTGACGGATA.

### Patients and Tissue Specimens

Primary HCC specimens along with complete clinical and pathological data were obtained from 255 HCC patients who underwent surgical resection at Sun Yat-sen University Cancer Center (SYSUCC), Guangzhou, China, between Jan 2001 and Dec 2007. The cohort consisted of 227 (89.0%) males and 28 (11.0%) females. The mean age was 47.9, ranging from 14.0 to 78.0. Postsurgical survival data were available for all 255 patients. Another 18 fresh HCC resection tissues and the corresponding adjacent liver tissues were collected for quantitative real-time PCR and western blot analysis. None of the patients had received adjuvant therapies before surgery. Tumor stage was defined according to the tumor-node-metastasis (TNM) classification of the American Joint Committee on International Union against Cancer. Tumor differentiation was assessed according to the Edmonson and Steiner grading system. The use of tissues for this study was approved by the Institute Research Medical Ethics Committee of SYSUCC. No informed consent (written or verbal) was obtained for use of retrospective tissue samples from the patients within this study, given that this was not deemed necessary by the Ethics Committee who waived the need for consent. All of the samples were anonymous.

### Tissue Microarray (TMA) Construction

TMA containing 255 HCC and adjacent nontumorous liver tissues were constructed. Briefly, all of the specimens were fixed in 4% formalin and embedded in paraffin. The corresponding histological H&E-stained sections were reviewed by a senior pathologist to mark out representative areas. Using a tissue array instrument (Beecher Instruments, Silver Spring, MD), each tissue core with a diameter of 0.6 mm was punched from the marked areas and re-embedded.

### Immunohistochemistry (IHC)

Formalin-fixed and paraffin-embedded HCC sections with a thickness of 4 µm were dewaxed in xylene and graded alcohols, hydrated, and washed in phosphate-buffered saline (PBS). After pretreatment in a microwave oven, endogenous peroxidase was inhibited by 3% hydrogen peroxide in methanol for 20 min, followed by avidin-biotin blocking using a biotin-blocking kit (DAKO, Germany). Slides were then incubated with Fibulin-3 antibody (1∶400, sc-33722) overnight in a moist chamber at 4°C, washed in PBS, and incubated with biotinylated goat anti-rabbit/mouse antibodies. Slides were developed with the Dako Liquid 3, ’3-diaminobenzidine tetrahydrochloride (DAB)+Substrate Chromogen System and counterstained with hematoxylin.

### Quantitative Real-time PCR (qRT-PCR)

Total RNA was extracted from paired HCC samples with Trizol reagent (BIOO Scientific Co., USA), following the manufacturer’s instructions. The mRNA was reverse transcribed to cDNA by M-MLV Reverse Transcriptase (Promega Inc., USA). The levels of Fibulin-3 and β-actin were measured by SYBR green-based real-time PCR using the Stratagene Mx3000P Real-Time PCR system. Primers were designed as follows: Fibulin-3, forward: 5′- CAGGACACCGAAGAAACCAT-3′ and reverse: 5′-GTTTCCTGCTGAGGCTGTTC-3′; and β-actin, forward: 5′-TGGCACCCAGCACAATGAA-3′ and reverse: 5′-CTAAGTCATAGTCCGCCTAGAAGC A-3′. Conditions were set as follows: one cycle of 95°C for 10 min, followed by 40 amplification cycles of denaturation at 95°C for 10 s, annealing at 60°C for 20 s and elongation at 72°C for 15 s. Using the comparative threshold cycle (2^−ΔCt^) method, the relative expressions of Fibulin-3 in HCC were normalized to endogenous β-actin.

### Western Blot

Cell or tissue lysates were boiled with 6X sodium dodecyl sulfate (SDS) loading buffer and then fractionated by SDS-PAGE. The proteins were transferred to PVDF membranes, incubated with a primary specific antibody for Fibulin-3 (1∶1000, Santa-Cruz Company, sc-33722) in 5% non-fat milk, and then incubated with a horse radish peroxidase (HRP)-conjugated anti-mouse secondary antibody. ECL detection reagent (Amersham Life Science, Piscataway, NJ, USA) was used to show the results.

### MTT

Cell viability was assessed by 3-(4, 5-dimethylthiazol-2-yl)-2, 5-diphenyltetrazo-lium bromide (MTT) assay. Briefly, 8×10^3^ cells transfected with scramble or Fibulin-3 siRNA were seeded into 96-well plates and incubated for 24 h. After adding 100 ml/well of MTT solution, the cells were incubated for another 2.5 h. Supernatants were then removed, and the formazan crystals were dissolved in 100 ml/well DMSO. The absorbance at 570/630 nm of each sample was measured using a multilabel plate reader (PerkinElmer). Three independent experiments were performed.

### Matrigel Invasion Assay

Cell invasion was performed using Millipore Biocoat Matrigel Invasion Chambers with 8 µM pore size (Millipore, Darmstadt, Germany), according to the manufacturer’s instructions.

### IHC Evaluation

Semi-quantitative IHC detection was used to determine the Fibulin-3 protein levels. Using the H-score method, we multiplied the percentage score by the staining intensity score. The percentage of positively stained cells was scored as “0” (0%), “1” (1%–25%), “2” (26%–50%), “3” (51%–75%), or “4” (76%–100%). Intensity was scored as “0” (negative staining), “1” (weak staining), “2” (moderate staining), or “3” (strong staining). For each case, 1000 cells were randomly selected and scored. The scores were independently decided by 2 pathologists (Dr. Y Cao and Dr. MY Cai).

### Selection of Cutoff Score

Receiver operating characteristic (ROC) curve analysis was employed to determine the cutoff score for tumors with high Fibulin-3 expression using the 0,1-criterion. For the outcomes being studied in HCC patients, sensitivity and specificity were plotted against the Fibulin-3 scores to generate various ROC curves. The count closest to the points of maximum sensitivity and specificity was selected as the cutoff value. Cases defined as having high Fibulin-3 expression had scores below or equal to the cutoff value, while cases defined as having low Fibulin-3 expression had scores above the cutoff value. To perform ROC curve analysis, clinicopathological features were dichotomized as follows: tumor multiplicity (single vs. multiple), tumor size (<5 cm vs. ≥5 cm), AFP level (<20 ng/ml vs. ≥20 ng/ml), tumor differentiation (well-moderate vs. poor-undifferentiated), stage (I+II vs. III+IV), vascular invasion (yes vs. no), relapse (yes vs. no) and survival status (dead vs. alive).

### Statistical Analysis

The statistical analyses were performed using the SPSS 16.0 software (SPSS, Chicago, IL, USA). ROC curve analysis was applied to determine the cutoff value for high expression of Fibulin-3 by the 0,1-criterion, and the areas under the curve (AUCs) were calculated. The Mann-Whitney U test was used for comparison between groups. The Wilcoxon matched paired test was used to determine the significance of Fibulin-3 expression in fresh HCC and normal liver tissues. The χ^2^ test was performed to analyze the correlation between Fibulin-3 expression and clinicopathological parameters. The Kaplan-Meier method (the log-rank test) was utilized for survival analysis and univariate analysis. Different independent analyses were performed depending on the selected population (overall population and various morphological and pathological subgroups). The Cox proportional hazards regression model was used to identify independent prognostic factors. *P*<0.05 (two-tailed) was considered to be statistically significant.

## Results

### Fibulin-3 Expression in HCC Cell Lines and Tissues by qRT-PCR and Western Blot

**Figure 1:**
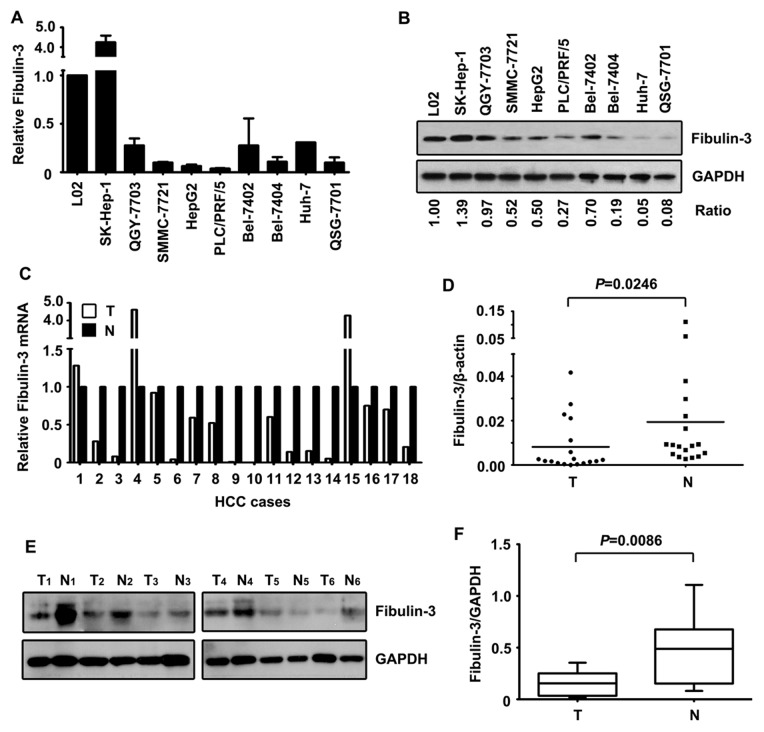
Expression of Fibulin-3 in HCC cell lines and fresh tissue samples by qRT-PCR and western blot. A. The mRNA levels of Fibulin-3 in immobilized liver cell line (L02) and HCC cell lines were determined using qRT-PCR. B. Protein levels of Fibulin-3 in HCC cell lines were detected by western blot. The ratio of Fibulin-3/GAPDH was indicated below. C. The mRNA levels of Fibulin-3 in 18 pairs of HCC and corresponding adjacent liver samples were examined. Relative Fibulin-3 mRNA was presented. D. Significance of alteration of Fibulin-3 mRNA was revealed by Wilcoxon matched paired test. E. Expressions of Fibulin-3 protein in 18 paired tissues were examined by western blot. Representative images of Fibulin-3 expression were presented. F. Relative intensity of Fibulin-3 normalized to GAPDH was calculated (n = 18).

First, we determined the expression of Fibulin-3 in HCC cell lines, using qRT-PCR and western blot. The results showed that Fibulin-3 was markedly decreased in HCC cell lines at both the mRNA (Fig. 1A) and protein (Fig. 1B) levels, compared with the expression in the immortalized liver cell line L-02.

This decrease in Fibulin-3 was further confirmed in 18 pairs of HCC fresh tissues. In 77.8% (14/18) of the samples, Fibulin-3 mRNA levels were much lower in HCC than normal tissues (Fig. 1C&D). Additionally, protein levels of Fibulin-3 were significantly reduced in tumorous tissues compared to those in adjacent nontumorous samples (Fig. 1E&F).

### Determination of Cutoff Value for Low Fibulin-3 Expression in HCC

**Figure 2:**
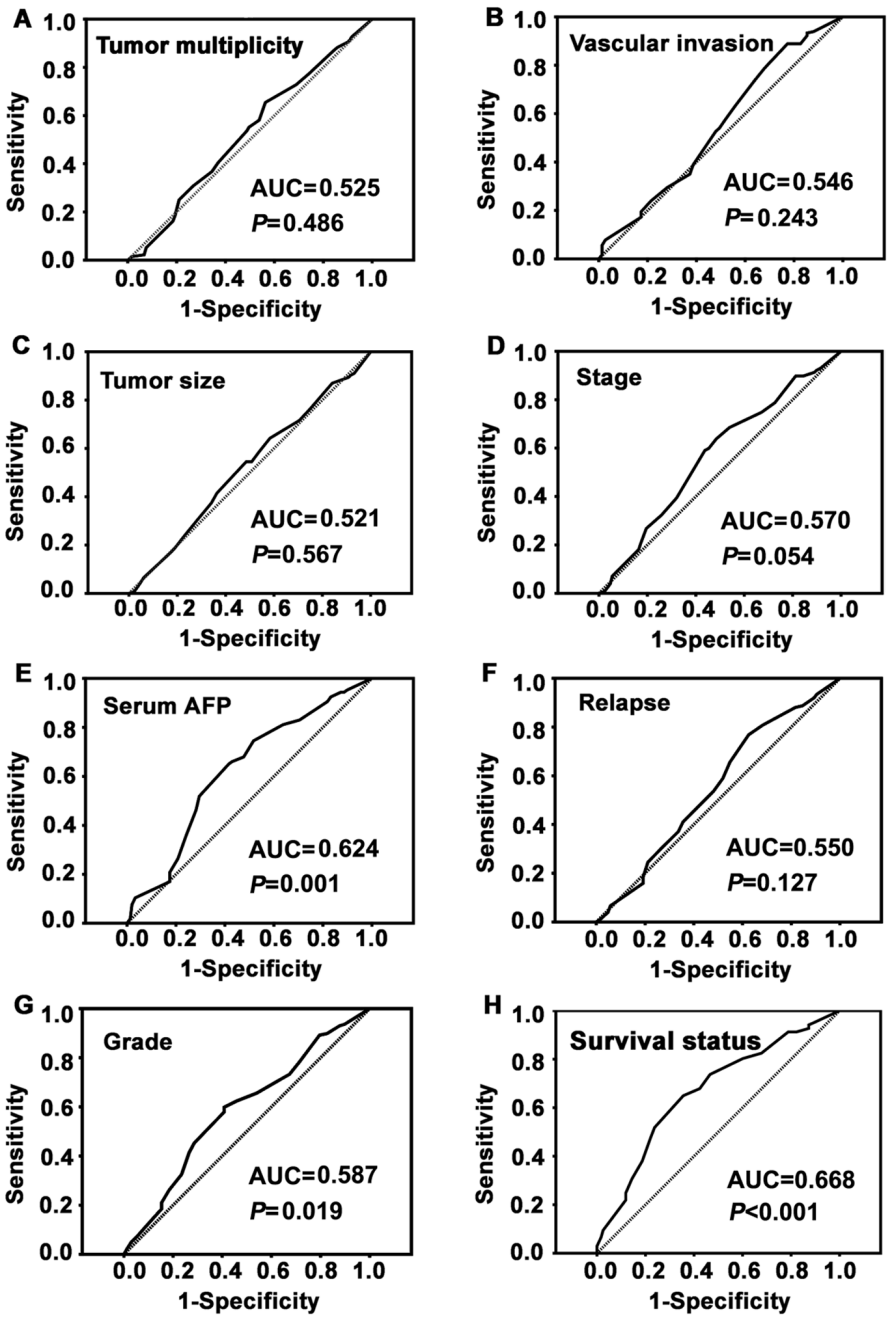
Determination of the cutoff value of low Fibulin-3 expression in HCC tissues by ROC curves. The sensitivity and 1-specificity were plotted for each clinical feature, such as tumor multiplicity, tumor size, serum AFP, pathological grade, clinical stage, vascular invasion, relapse and survival status. The areas under the curve (AUCs) and the *P* values were indicated.

To determine an optimal cutoff value to identify low Fibulin-3 expression, an ROC curve was utilized according to the results of the IHC evaluation. The ROC curve for survival status possessed the smallest distance (0.0, 1.0), indicating that Fibulin-3 expression has the greatest prognostic ability (maximum sensitivity and specificity) for survival status (Fig. 2). Therefore, the score of 6.75 was chosen as the cutoff value for low Fibulin-3 expression.

### Association between Fibulin-3 Expression and Clinicopathological Features

**Figure 3:**
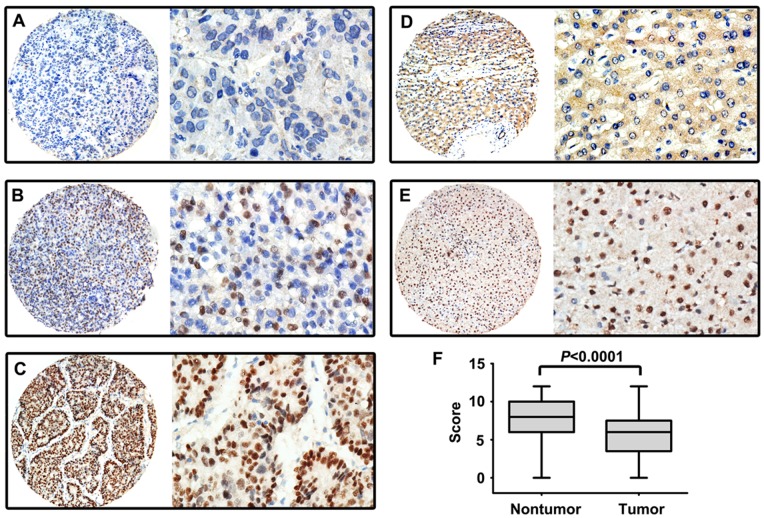
Expression of Fibulin-3 in HCC tissues by IHC. Micrographs showed weak (A), moderate (B), and strong (C) staining of Fibulin-3 in HCC, as well as low (D) and high (E) expression of Fibulin-3 in normal liver tissues. (Left panel: magnification ×100; Right panel: magnification ×400.) F. Reproducibility of the measurement in all 255 patients was calculated using the Wilcoxon matched paired test.

A cohort comprising 255 HCC cases was collected to construct a TMA to determine the expression pattern of Fibulin-3 in HCC. The results of the TMA-based IHC showed that Fibulin-3 was mainly expressed in the nucleus of tumor (Fig. 3A–C) and normal liver cells (Fig. 3D&E). The overall expression of Fibulin-3 was revealed to be significantly downregulated in HCC tissues compared to nontumorous tissues (Fig. 3F). Furthermore, Fibulin-3 was reduced in tumorous tissues in 67.1% (171/255) of cases compared to the corresponding adjacent nontumorous tissues.

We next investigated the relationship between Fibulin-3 expression and clinicopathological variables of HCC patients. The statistical analysis showed that low Fibulin-3 expression was significantly correlated with poor differentiation (*P* = 0.008), advanced stage (*P* = 0.014), and high AFP serum levels (*P*<0.001). However, there were no statistical connections between Fibulin-3 expression and the other clinicopathological parameters, including age, gender, HBsAg, cirrhosis, tumor multiplicity, tumor size, vascular invasion, and disease relapse (*P*>0.05, Table 1).

**Table 1:** Correlation between the clinicopathologic variables and Fibulin-3 expression in HCC.

Variable	Fibulin-3 protein				
	All cases	Low expression	High expression	?2	*P* value^a^
Age (years)^b^				1.177	0.278
<47.9	133	69 (51.9%)	64 (48.1%)		
≥47.9	122	55 (45.1%)	67 (54.9%)		
Gender				0.024	0.878
Male	227	110 (48.5%)	117 (51.5%)		
Female	28	14 (50%)	14 (50%)		
HBsAg				0.127	0.722
Positive	222	105 (48.2%)	115 (51.8%)		
Negative	33	17 (51.5%)	16 (48.5%)		
AFP (ng/ml)				13.673	**<0.001**
<20	106	37 (34.9%)	69 (65.1%)		
≥20	149	87 (58.4%)	62 (41.6%)		
Cirrhosis				0.170	0.680
Yes	184	88 (47.8%)	96 (52.2%)		
No	71	36 (50.7%)	35 (49.3%)		
Tumor size (cm)				0.913	0.339
<5	123	56 (45.5%)	67 (54.5%)		
≥5	132	68 (51.5%)	64 (48.5%)		
Tumor multiplicity				0.619	0.431
Single	136	63 (46.3%)	73 (53.7%)		
Multiple	119	61 (51.3%)	58 (48.7%)		
Differentiation				7.100	**0.008**
Well-Moderate	157	66 (42.0%)	91 (58.0%)		
Poor-Undifferentiated	98	58 (59.2%)	40 (40.8%)		
Stage				5.978	**0.014**
I–II	127	52 (40.9%)	75 (59.1%)		
III–IV	128	72 (56.3%)	56 (43.7%)		
Vascular invasion				0.484	0.487
Yes	75	39 (52.0%)	36 (48.0%)		
No	180	85 (47.2%)	95 (52.8%)		
Relapse				0.764	0.382
Yes	104	54 (51.9%)	50 (48.1%)		
No	151	70 (46.4%)	81 (53.6%)		

a Chi-squared test;

b Mean age; AFP, alpha-fetoprotein; HBsAg, hepatitis B surface antigen.

### Correlation of Fibulin-3 Expression with Prognosis of HCC Patients

**Figure 4:**
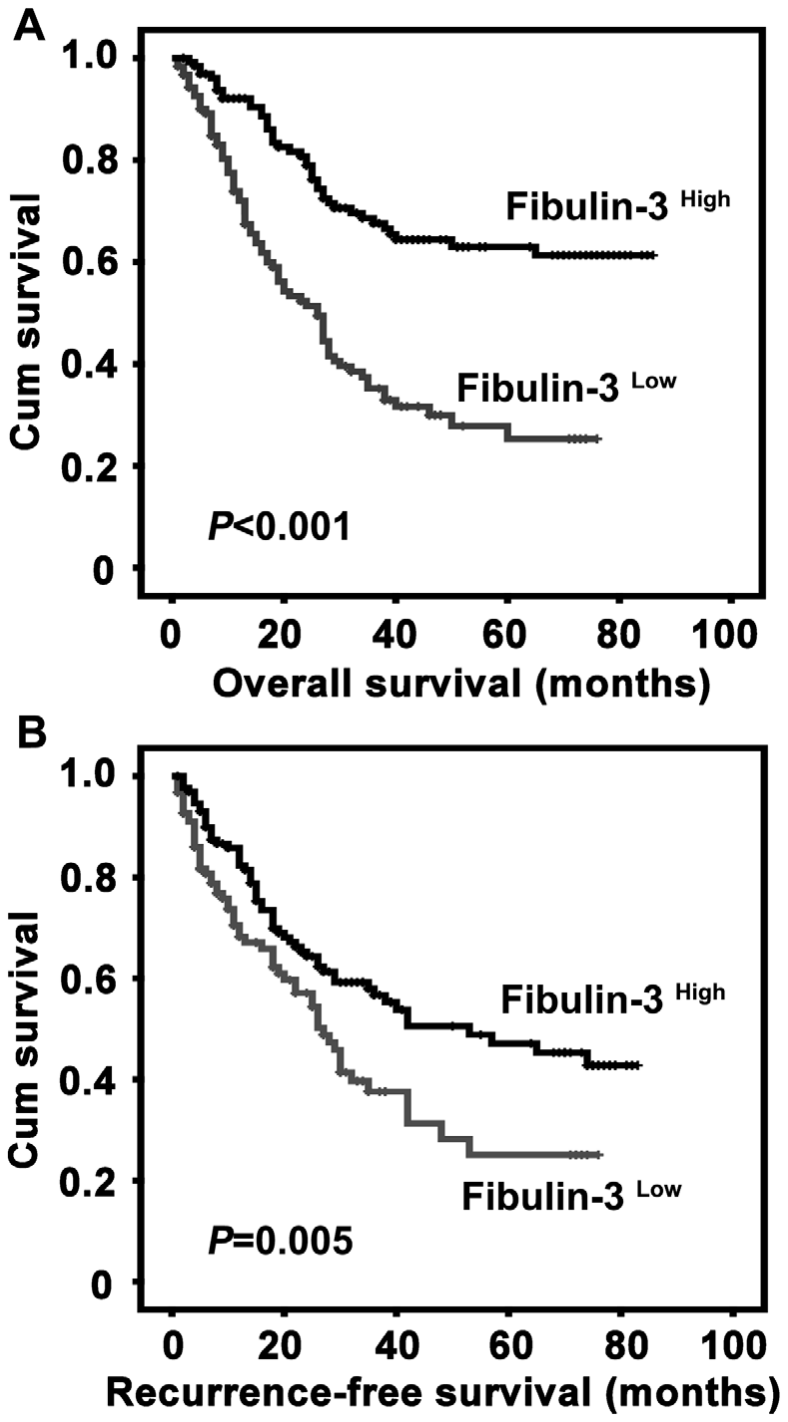
Association of low Fibulin-3 expression in HCC tissue and unfavorable overall survival and recurrence-free survival by Kaplan-Meier survival analysis. Probabilities of overall survival (A) and recurrence-free survival (B) of 255 total HCC patients were analyzed using Kaplan-Meier survival analysis (log-rank test).

To determine the value of Fibulin-3 for the prognosis of postsurgical HCC patients, Kaplan-Meier survival analyses were conducted. Survival data were available for all 255 patients. The average survival time was 24.5 months for patients with low Fibulin-3 expression and 40.5 months for patients with high Fibulin-3 expression. The results indicated that patients with low Fibulin-3 expression had much shorter survival times (*P*<0.001) (Fig. 4A) and had a higher tendency of disease recurrence (*P* = 0.005) (Fig. 4B).

**Figure 5:**
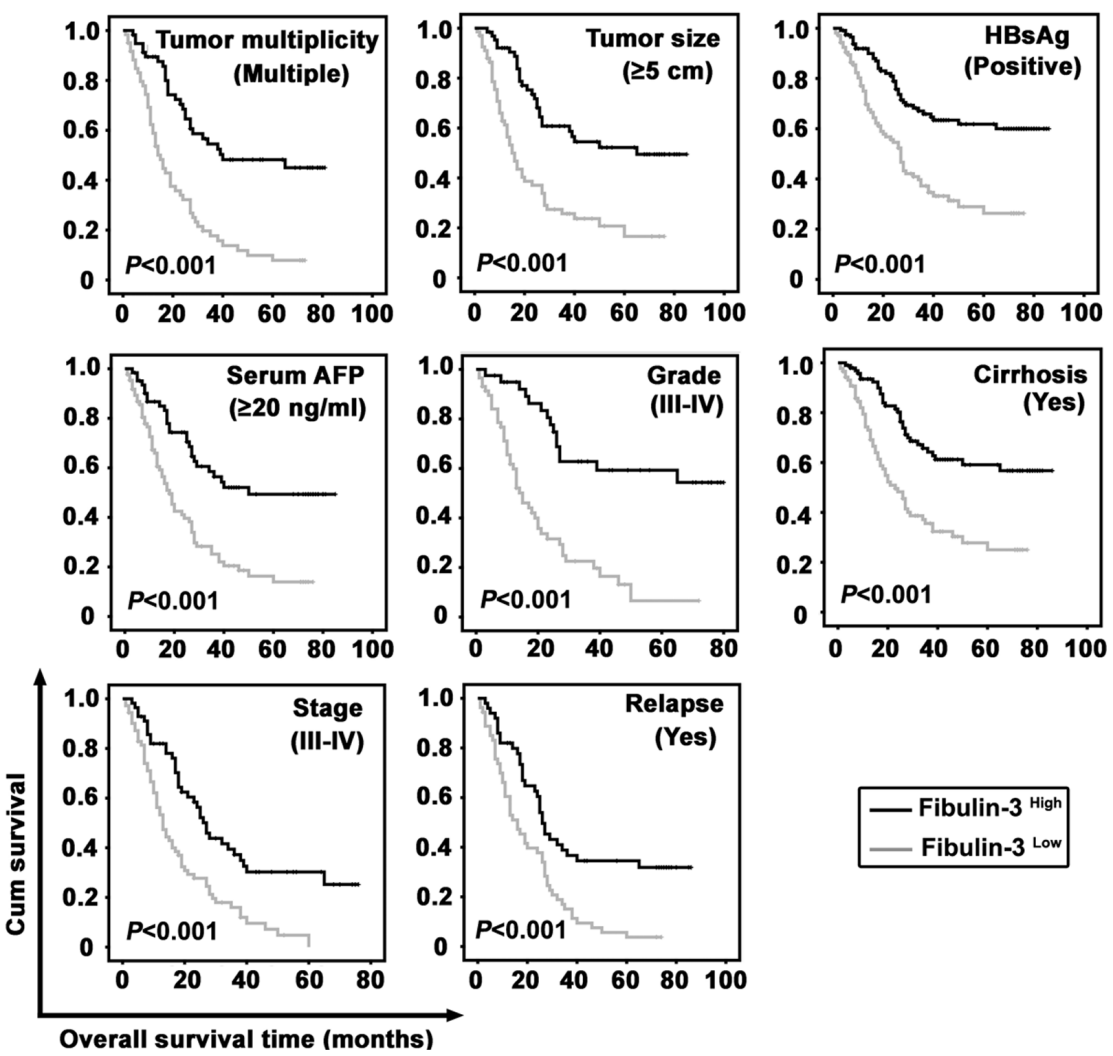
Correlation of Fibulin-3 expression with overall survival in morphologic and pathological HCC subgroups. Survival analyses were performed in subgroups divided by the factors that contribute to poorer outcomes of HCC patients, using Kaplan-Meier survival analysis (log-rank test).

The prognostic value of Fibulin-3 was further confirmed by stratified survival analysis. Results showed that low expression of Fibulin-3 was closely connected with poor overall survival after surgical resection in 8 subgroups classified by the factors contributing to poorer outcome in HCC patients (*P*<0.001 for all of the groups) (Fig. 5). The effect of Fibulin-3 on the recurrence-free survival rates of the 8 subgroups was also determined (Fig. S1).

### Univariate and Multivariate Analyses of Prognostic Variables in HCC Patients

To test whether the cohort in the present study was representative, univariate analysis was performed. Results showed that Fibulin-3, as well as tumor size, serum AFP level, tumor multiplicity, clinical stage, vascular invasion, tumor differentiation and relapse, were responsible for the overall and recurrence-free survival of HCC patients (Table 2&3).

**Table 2:** Univariate and multivariate analyses of prognostic factors of overall survival.

Variable	All cases	Univariate			Multivariate	
		Mean	Median	*P* value	HR (95% CI)	*P* value
Age (years)^a^				0.804		
<47.9	133	50.2	39.0			
≥47.9	122	48.6	38.0			
Gender				0.408		
Male	227	49.2	38.0			
Female	28	52.5	NR			
HBsAg				0.644		
Positive	222	50.2	40.0			
Negative	33	44.8	38.0			
Cirrhosis				0.477		
Yes	184	48.7	38.0			
No	71	52.3	NR			
AFP (ng/ml)				**<0.001**	1.753 (1.122-2.739)	**0.014**
<20	106	65.3	NR			
≥20	149	38.9	27.0			
Tumor size (cm)				**<0.001**	1.121 (0.752-1.671)	0.575
<5	123	59.6	NR			
≥5	132	41.6	27.0			
Tumor multiplicity				**<0.001**	1.074 (0.668-1.727)	0.767
Single	136	63.1	NR			
Multiple	119	35.6	24.0			
Differentiation				**<0.001**	1.059 (0.723-1.549)	0.770
Well-Moderate	157	57.1	NR			
Poor-Undifferentiated	98	36.9	25.0			
Stage				**<0.001**	5.199 (2.837-9.527)	**<0.001**
I–II	127	73.8	NR			
III–IV	128	26.7	18.0			
Vascular invasion				**<0.001**	1.197 (0.785-1.826)	0.402
Yes	75	28.4	18.0			
No	180	58.6	NR			
Relapse				**<0.001**	2.250 (1.464-3.457)	**<0.001**
Yes	104	30.9	24.0			
No	151	65.1	NR			
Fibulin-3				**<0.001**	0.468 (0.315-0.695)	**<0.001**
Low expression	131	34.1	26.0			
High expression	124	62.0	NR			

a Mean age; NR, not reached; HbsAg, hepatitis B surface antigen; AFP, alpha-fetoprotein; HR, hazard ratio; CI, confident interval.

**Table 3:** Univariate and multivariate analyses of prognostic factors of recurrence-free survival.

Variable	All cases	Univariate			Multivariate	
		Mean	Median	*P* value	HR (95% CI)	*P* value
Age (years)^a^				0.725		
<47.9	133	43.2	30.0			
≥47.9	122	42.9	36.0			
Gender				0.459		
Male	227	44.7	35.0			
Female	28	33.2	42.0			
HBsAg				0.291		
Positive	222	43.0	35.0			
Negative	33	47.9	42.0			
Cirrhosis				0.379		
Yes	184	41.6	30.0			
No	71	48.5	42.0			
AFP (ng/ml)				**<0.001**	1.444 (0.974-2.142)	0.067
<20	106	53.0	57.0			
≥20	149	36.0	26.0			
Tumor size (cm)				**<0.001**	1.276 (0.857-1.899)	0.230
<5	123	53.2	NR			
≥5	132	35.7	22.0			
Tumor multiplicity				**0.007**	1.122 (0.748-1.682)	0.579
Single	136	49.3	42.0			
Multiple	119	36.7	26.0			
Differentiation				**0.001**	1.202 (0.819-1.763)	0.348
Well-Moderate	157	49.6	53.0			
Poor-Undifferentiated	98	32.1	26.0			
Stage				**<0.001**	2.262 (1.319-3.879)	**0.003**
	127	61.5	NR			
III–IV	128	24.5	18.0			
Vascular invasion				**<0.001**	2.665 (1.718-4.133)	**<0.001**
Yes	75	19.6	15.0			
No	180	57.2	NR			
Fibulin-3				**0.005**	0.676 (0.466-0.979)	**0.038**
Low expression	131	34.3	27.0			
High expression	124	49.5	53.0			

a Mean age; NR, not reached; HbsAg, hepatitis B surface antigen; AFP, alpha-fetoprotein; HR, hazard ratio; CI, confident interval.

Multiple Cox regression analysis was further utilized to evaluate the independent prognostic value of Fibulin-3. Results revealed that Fibulin-3 was an independent prognostic marker for overall survival (Hazard Ratio (HR) 0.468, *P*<0.001) and recurrence-free survival (Hazard Ratio (HR) 0.676, *P* = 0.036) (Table 2&3).

### Increase of Cell Proliferation and Invasion by Fibulin-3 siRNA

**Figure 6:**
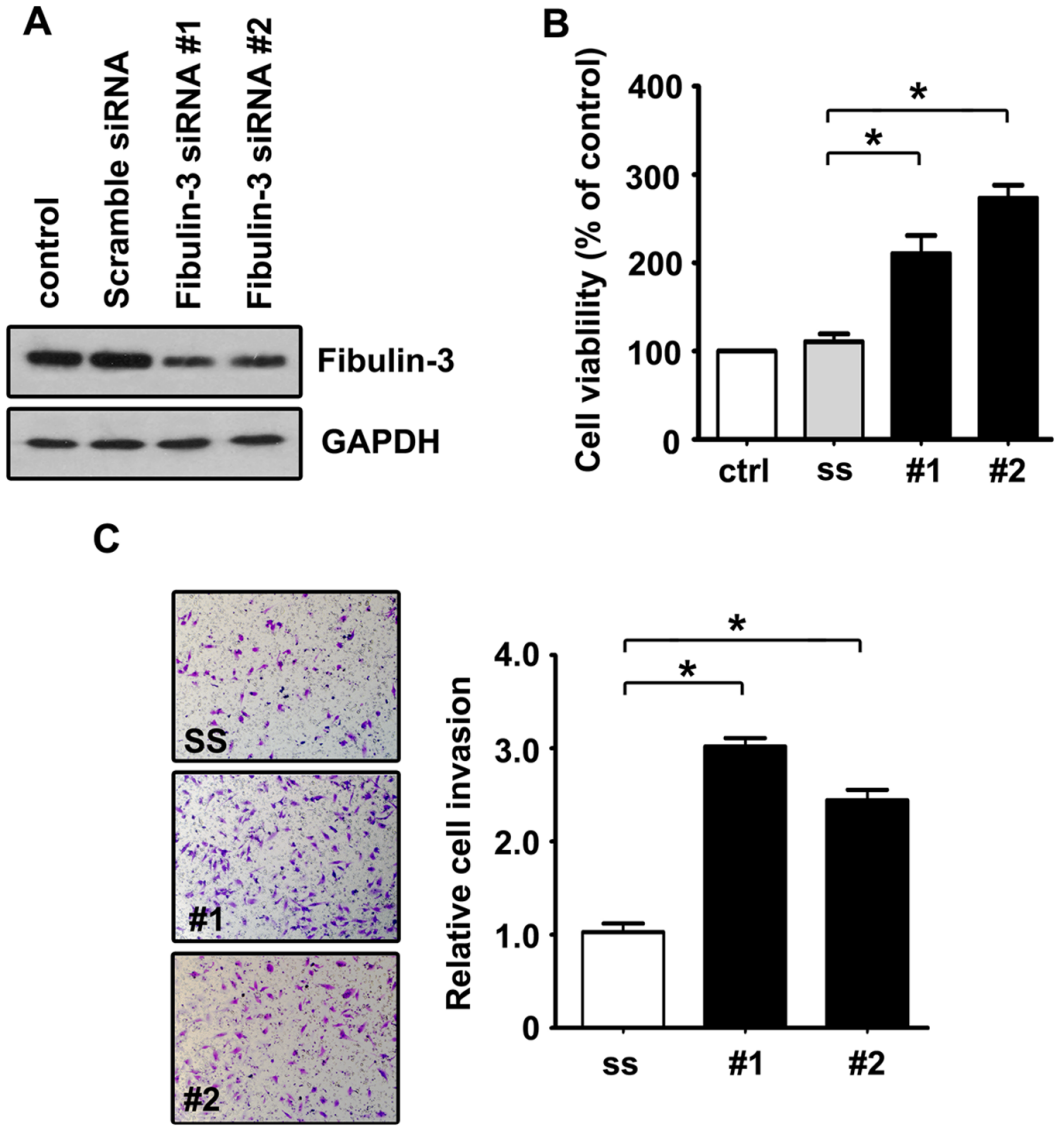
Promotions of cell proliferation and invasion by Fibulin-3 siRNA in QGY-7703 cells. A. Fibulin-3 siRNA noticeably downregulated expression of Fibulin-3 protein. Scramble and Fibulin-3 siRNA were transfected into QGY-7703 cells for 24 h. Relative Fibulin-3 expressions were detected by western blot. B. Fibulin-3 siRNA significantly increased cell viability in HCC cells. Cells transfected with scramble siRNA (ss) and Fibulin-3 siRNA (#1 and #2) were seeded into 96-well plates and cultured for 24 h. The cell viabilities were determined using MTT assays. The data are means ± SD of three independent experiments, *P<0.05. C. Fibulin-3 siRNA promoted cell invasion *in vitro*. QGY-7703 cells were seeded into transwell chambers coated with Matrigel. After 24 h, invaded cells stained by 0.1% crystal violet were counted. The data are means ± SD of three independent experiments, *P<0.05.

We next examined whether a decrease in Fibulin-3 affected HCC progression. Two Fibulin-3 siRNAs were proven to effectively downregulate the expression of Fibulin-3 (Fig. 6A). The knockdown of Fibulin-3 in QGY-7703 cells led to a reduction in cell viability (Fig. 6B), as indicated by MTT assay. The effect of Fibulin-3 on cell invasion was further investigated using a Matrigel invasion assay. Results showed that Fibulin-3 inhibition significantly increased serum-induced transwell migration of QGY-7703 cells by 3.04 and 2.45-fold in siRNA #1 and siRNA#2 groups, respectively (Fig. 6C).

## Discussion

HCC is a heterogeneous cancer with very high mortality. Searching for valuable biomarkers for HCC diagnosis and prognostic prediction has been attracting an increasing amount of interest. Plenty of proteins, such as SIRT3 [24], DKK1 [25] and PLK4 [26], have been shown to have clinical significance for predicting HCC prognosis. In addition, other signatures of cancer, including DNA methylation, circulating tumor cells and histone modification, have been attracting the attention of researchers for use in determining the nature of HCC. In this study, we aimed to investigate the expression and the prognostic value of Fibulin-3 in a large cohort of HCC patients.

Our data showed that the Fibulin-3 protein level was decreased in HCC patients and was associated with unfavorable prognosis. It is noteworthy that Fibulin-3 acts as an independent prognostic biomarker in HCC. Fibulin-3 expression was inversely correlated with both overall and recurrence-free survival of HCC patients. Examination of Fibulin-3 expression in HCC may aid in the development of new therapeutic strategies. In agreement with our data, decreased levels of Fibulin-3 were observed in other human cancers. For example, Tong et al. reported that Fibulin-3 was downregulated in colorectal cancer and was associated with poor prognosis [18]. Hwang et al. demonstrated that reduction of Fibulin-3 was associated with tumor progression and poor prognosis in nasopharyngeal carcinomas [22]. Sadr-Nabavi et al. showed that reduction of Fibulin-3 in sporadic breast cancer was correlated with poor prognosis [20]. Surprisingly, Fibulin-3 was also found to be upregulated in other cancers. Song et al. showed that the overexpression of Fibulin-3 in cervical carcinoma was an indicator of poor survival [16]. Increased expression of Fibulin-3 was observed in malignant glioma [17]. Recently, the upregulation of plasma Fibulin-3 was proven to be of clinical significance for the diagnosis of pleural mesothelioma [14]. In the present study, low Fibulin-3 expression was related to poor differentiation and advanced stages of HCC, suggesting Fibulin-3 may be involved in HCC progression. Although promoter methylation contributes to Fibulin-3 downregulation in human cancers, including colorectal cancer, lung cancer and HCC, the detailed mechanism by which Fibulin-3 is downregulated in HCC requires future investigation.

Paradoxical effects of Fibulin-3 on tumor progression have been reported. The overexpression of Fibulin-3 in Hela cells promotes angiogenesis, proliferation and invasion by increasing the expression of VEGF [27]. In pancreatic adenocarcinomas, Fibulin-3 binds EGFR (competitive to EGF) causing autophosphorylation of EGFR at Tyr-992 and Tyr-1068 and the subsequent phosphorylation of AKT at Thr-308 and ERK at Thr-202 and Tyr-204 and, thus, accelerates pancreatic adenocarcinoma growth [28]. Fibulin-3 promoted glioma growth by promoting Notch-1 cleavage and upregulating the active Notch-1 intracellular domain (NICD) to reduce apoptosis [29]. Interestingly, an opposite effect of Fibulin-3 in glioma was also shown; overexpression of Fibulin-3 inhibited malignant glioma growth by suppressing EGFR-AKT signaling [30]. Antitumor activity of Fibulin-3 was also reported in other cancers. For example, Albig et al. showed that Fibulin-3 abolished angiogenic activities and sprouting in MB114 cells by decreasing the expression of MMP-2 and MMP-3 and increasing the expression of TIMP-1 and TIMP-3. [31]. Kim and colleagues observed that enforced expression of Fibulin-3 in A549 non-small cell lung cancer (NSCLC) cells attenuated cell invasion by reducing expressions of MMP-2 and MMP-7 [32]. Hwang et al. reported that Fibulin-3 inhibited nasopharyngeal carcinoma cell migration and invasion by decreasing the phosphorylation of AKT at Ser-473 [22]. Furthermore, Fibulin-3 sensitized pancreatic cancer cells to a PI3K/mTOR inhibitor by interacting with p27^Kip1^ [33]. In the present study, Fibulin-3 was found to be decreased in HCC cell lines and tissue samples. Low Fibulin-3 expression was associated with malignant parameters and unfavorable prognosis. *In vitro* experiments demonstrated that knockdown of Fibulin-3 resulted in an increase in cell viability and invasion. Collectively, our data suggest Fibulin-3 as a tumor suppressor in HCC.

In most cases, the biological functions of proteins depend on their cellular localization. Fibulin-3 is a member of the fibulin family of extracellular glycoproteins, suggesting Fibulin-3 should be localized to the cytoplasm. It has been reported that Fibulin-3 interacts with extracellular matrix protein 1 (ECM1) [34] and tissue inhibitor of metalloproteinase 3 (TIMP-3) [35] to localize to the basement membrane. However, nuclear Fibulin-3 was also depicted in malignant gliomas [17], pleural mesothelioma [14] and nasopharyngeal carcinomas [22]. In the present study, Fibulin-3 was observed in both the cytoplasm and nucleus of HCC cells. As Fibulin-3 lacks a nuclear localization signal, the mechanism of translocation of Fibulin-3 into the nucleus and its nuclear function remain to be determined.

In summary, our data provide comprehensive understanding that Fibulin-3 is remarkably downregulated in HCC. Reduction of Fibulin-3 was associated with tumor differentiation, clinical stage and serum AFP level. Low Fibulin-3 expression predicts lower overall survival and recurrence-free survival. The knockdown of Fibulin-3 in HCC cells resulted in cell proliferation and invasion. Taken together, although the detailed mechanism remains unclear, Fibulin-3 functions as a tumor suppressor in HCC and is of clinical significance in predicting the postsurgical prognosis of patients who suffer from this deadly disease.

## Supporting Information

Figure S1 Relationship of Fibulin-3 expression and recurrence-free survival in HCC subgroups. Survival analysis was performed in subgroups according to the factors that are attributed to worse outcome of HCC patients, using Kaplan-Meier survival analysis (log-rank test). (TIF)
