# Supplementary figures and images for: Decrease of Fibulin-3 in Hepatocellular Carcinoma Indicates Poor Prognosis
Source: PLoS One. 2013 Aug 1;8(8):e70511. doi: 10.1371/journal.pone.0070511 (PMC3731361; doi:10.1371/journal.pone.0070511)

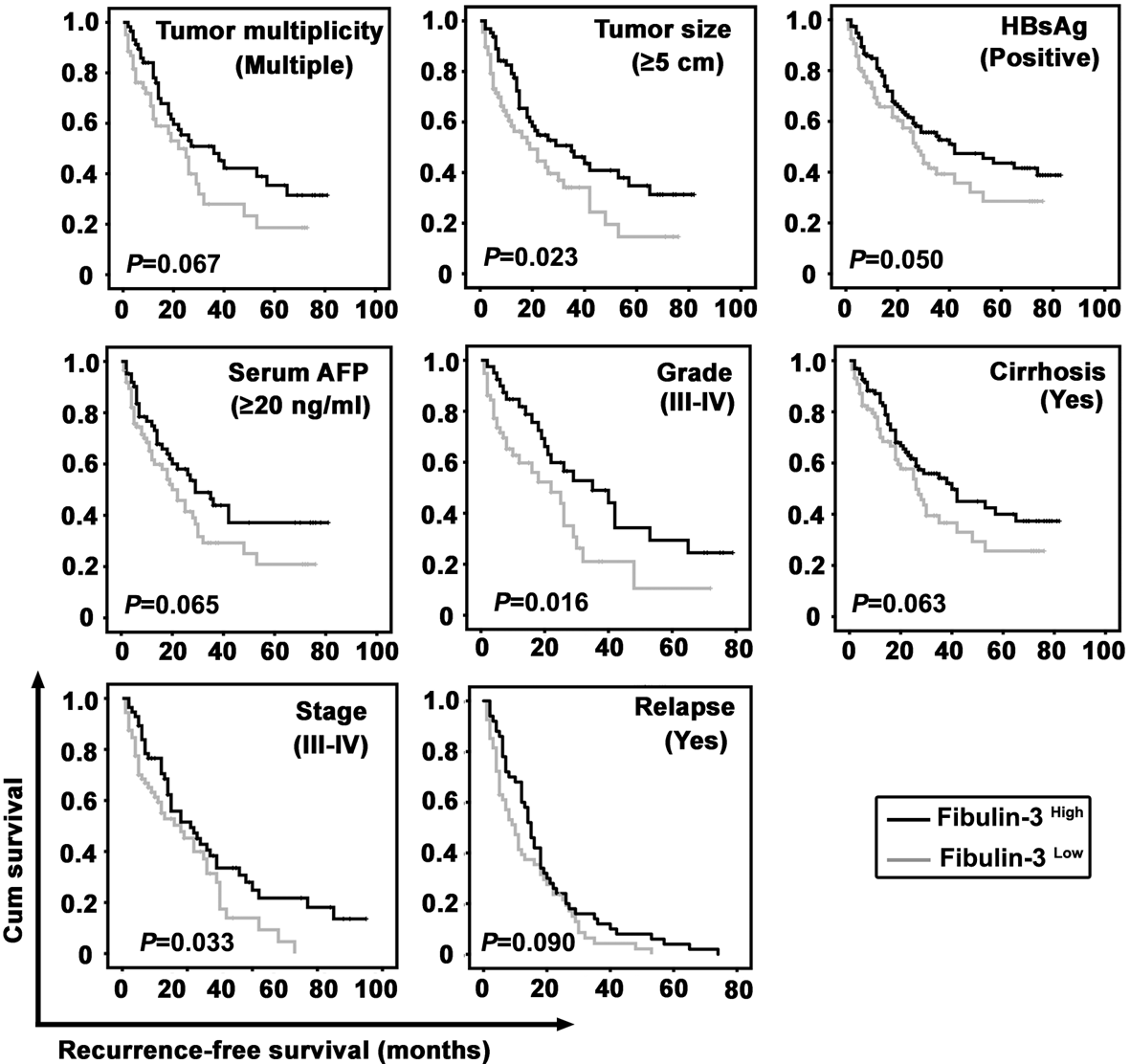

Supplement: Figure S1 — (TIF) [file pone.0070511.s001.tif]
